# Impact of vaccine coverage and disruption to health services on COVID-19 in Ukraine

**DOI:** 10.1038/s41598-024-57447-7

**Published:** 2024-06-26

**Authors:** Valentina Costantino, Chandini R. MacIntyre

**Affiliations:** 1 The Biosecurity Program, The Kirby Institute, University of New South Wales, High street, Kensington, Sydney, Australia; 2 College of Health Solutions, Arizona State University, Tempe, AZ USA; 3 Watts College of Public Affairs and Community Solutions, Arizona State University, Tempe, AZ USA

**Keywords:** Coronavirus, Modelling, Infectious diseases, Outbreak response, Ukraine, Masks, Vaccination, Infectious diseases, Vaccines, Diseases, Health care

## Abstract

COVID-19 surveillance in Ukraine ceased after the Russian invasion of the country in 2022, on a background of low vaccination rates of 34.5% for two doses at this time. We conducted a modelling study to estimate the epidemic trajectory of SARS-COV-2 in Ukraine after the start of the war. We use a COVID-19 deterministic Susceptible-Exposed-Infected-Recovered (SEIR) model for Ukraine to estimate the impact of increased vaccination coverage and masking as public health interventions. We fit the model output to case notification data between 6 January and 25 February 2022, then we forecast the COVID-19 epidemic trajectory in different scenarios of mask use and vaccine coverage. In the best-case scenario, 69% of the Ukrainian population would have been infected in the first half of 2022. Increasing mask use from 50 to 80% reduces cases and deaths by 17% and 30% respectively, while increasing vaccination rates to 60% and 9.6% for two and three doses respectively results in a 3% reduction in cases and 28% in deaths. However, if vaccination is increased to a higher coverage of 80% with two doses and 12.8% with three, or mask effectiveness is reduced to 40%, increasing vaccination coverage is more effective. The loss of health services, displacement, and destruction of infrastructure will amplify the risk of COVID-19 in Ukraine and make vaccine programs less feasible. Masks do not need the health infrastructure or cold-chain logistics required for vaccines and are more feasible for rapid epidemic control during war. However, increasing vaccine coverage will save more lives. Vaccination of refugees who have fled to other countries can be more feasibly achieved.

## Introduction

COVID-19 vaccination rates in Ukraine were 34.5% for two doses and 1.7% for 3 doses at the time of the Russian invasion of the country in late February 2022^1^. The conditions of war include internal displacement, homelessness and injuries. A large number of internally displaced people may gather in underground subways or other buildings for shelter, with such gatherings increasing the risk of SARS-COV-2 transmission. In addition, loss of access to health services, destruction of hospitals, and disruption to public health vaccination programs may amplify the impact of COVID-19 in Ukraine^2^.

It has been estimated that the Omicron variant of SARS-COV-2, which was dominant when the war began in Ukraine, has a basic reproduction number (R0) over 3 times higher^3^ than the Delta variant. Increased transmissibility may also be due to immune evasion rather than an intrinsic increase in R0^4^. Another study showed that the R0 for Omicron may be similar to Delta, and the increased transmission is mostly due to immune evasion, waning of vaccine protection, and reduction in the latent period^5–7^. The vaccines used in Ukraine include Pfizer, Sinovac, AstraZeneca, and Moderna, in the order of doses utilised. The Sinovac vaccine has a lower effectiveness than other vaccines^8^, with an estimated 64% effectiveness after two doses^9^. Therefore, the overall vaccine protection as a function of both coverage, number of doses, and vaccine efficacy in Ukraine is low. Furthermore, immunity following infection with the Omicron variant of the SARS-CoV-2 virus is lower than from Delta, and also about only one-third of the immunity provided by vaccination^10^. The continuous evolution of SARS-COV-2 has led to a rapid and simultaneous emergence of several variants, associated with increased immune escape and antibody evasion^11^. The Omicron BA.1 variant caused a large global wave of the pandemic in early 2022^12^ and was then replaced by the BA.2 variant. This had an even greater growth advantage, showing an increased probability of transmission per contact of 4%^13^. Since 2022, the dominant strains at different times were BA.5, followed by BQ.1, BQ.1.1, BA.4.6, and XBB variants^14,15^, with a similar epidemiology in Europe^16^. Immunity from infection, like vaccine-induced immunity, wanes, so in a population with low vaccination rates, reinfections may be common^5^. Genomic surveillance is unlikely to be occurring in Ukraine during the war, so the dominant variant is unknown. All COVID-19 surveillance data from Ukraine ceased on March 3, 2022, five days after the Russian invasion of the country. The start of the war likely caused the vaccination rollout to cease and impacted health system capacity. There are few studies on epidemic control during war. Furthermore, the effect of a conflict on infectious disease transmission is difficult to estimate in the absence of surveillance data. Vaccines and masks are two available mitigations for COVID-19, implementation of which may have variable feasibility in a war zone. We sought to model the COVID-19 epidemic in Ukraine following the start of the war with Russia, to inform feasible disease control policies^17,18^.

### Aims

To estimate the epidemic trajectory of SARS-CoV-2 in Ukraine after the start of the war and compare the effectiveness of increasing mask use and vaccine coverage respectively.

## Methods

### Model description

A deterministic Susceptible-Exposed-Infected-Recovered (SEIR) model of SARS-CoV-2 transmission in Ukraine was used to estimate the epidemic trajectory and influence of vaccination coverage and mask use on epidemic control. We used age-specific parameters for Ukraine to build a model and included the effect of estimated government restrictions (restriction index) on transmissions for each policy implemented from the Blavatnik School of Government restrictions tracker^17,18^.

**Figure 1:**
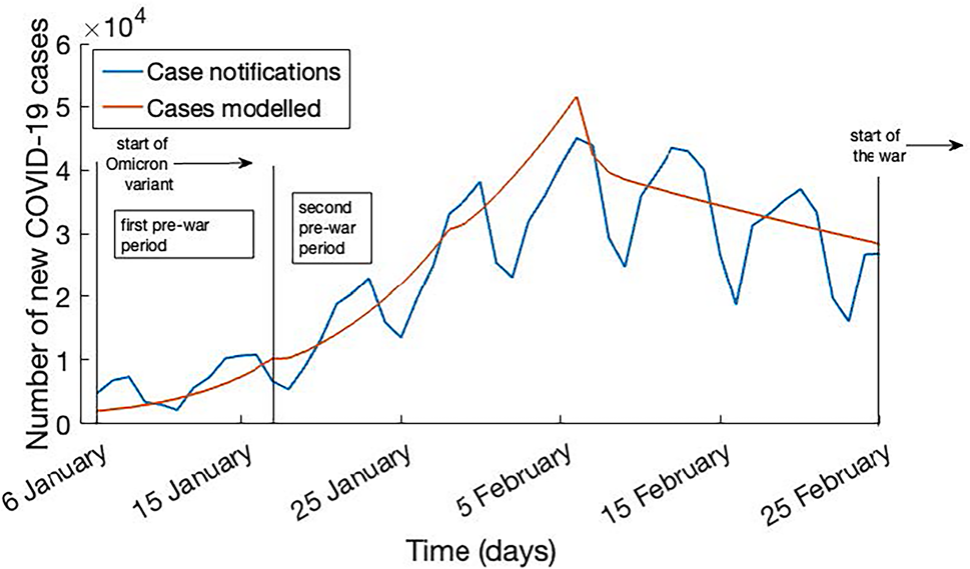
Model fit to clinical case notification data (COVID-19 incidence notification data) between 6 January 2022 and 25 February 2022, with masks 60% effective. The vertical lines represent the dates of policy changes. 6 January is the start of the Omicron wave; 6–17 January is the first pre-war period with the implemented restrictions policy^17,18^; 18–25 January is the second pre-war period where the reduction in contacts increases with the additional policy of closure of public transports and enforced mask use in all public spaces^17,18^; 25 February represent the start of the war, where vaccine rollout and contact tracing stops.

We use a deterministic compartmental model for disease transmission of COVID-19 based on a published and peer-reviewed model^19^. The model was built using MATLAB 2021b and adapted for the Ukraine population and the Omicron BA.2 variant of concern which were dominant in early 2022. The expanded SEIR model is based on a system of ordinary differential equations and moves the population through disease epidemiological stages and response stages as described in Appendix section 1 and shown in Fig. 1S in the Appendix. The differential equations are shown in Appendix section 2.

### Model parameters and data

The parameters (Table 1) reflect the transmission of the BA.2 Omicron variant which was the circulating strain at the time COVID-19 surveillance ceased, with a latent period reduced to 3 days. The infectious period is divided into 2 pre-symptomatic days and 7 symptomatic days^3,4,19^. We use R0 of 6^5–7^. In Ukraine four different vaccines were used, Pfizer, Sinovac, AstraZeneca (AZ), and Moderna. It has been shown that all vaccines have a reduced effectiveness against symptomatic infection for the Omicron variant^20^. As the combination of Pfizer and AZ represents just over 60% of the total doses used, in this study we used the estimated vaccine effectiveness of Pfizer and AZ against Omicron. Vaccine effectiveness against Omicron infection^21^ is assumed to be 5.9% and 34.2% after 6 months from vaccination following two doses of AZ and Pfizer respectively in adults. We used 71.4% and 75.5% protection against symptomatic infection^21^ for AZ and Pfizer recipients respectively following the Pfizer booster dose^20^. For hospitalization, ICU, and death rates, available European estimates (for EU/EEA countries, with the data collected from The European Surveillance System (TESSy) or the GISAID EpiCoV database) are 1.14%, 0.16%, and 0.06% respectively overall, due to the Omicron variant^22^. However, this estimation does not account for vaccination status, and Ukraine has very low vaccination coverage^1^ compared to the rest of Europe. Therefore, we used estimates of hospitalization, ICU, and death rates from the US^23^, which are available by vaccination status. Due to the lack of age-specific data on hospitalization, ICU, and deaths, we used the age distributions of the Delta variant from a previous study^19,24^, based on the COVID-19–Associated Hospitalization Surveillance Network (COVID-NET) data on 1482 people hospitalized in March 2022 in the US.

**Table 1:** Model parameters.

Symbol	Definition	Value				Source
R0	Basic reproductive number	6				^5–7^
$\theta$	Percentage of symptomatic people isolated	90%				^19^
ρ	Contacts traced for each case	60% (6-17 January) 45% (18 January-5 February) 20% (6-25 February) 0% following the 25 February				^17,18^
${q}_{1}$	Duration of quarantine for contacts traced isolation for symptomatic	7				
${d}_{0}$	Latent duration not infectious	1 day				
d	Duration in Infectious period before being traced	1 day				
${d}_{1}$	Pre symptomatic infectious duration	2 days				^19^
$d+{d}_{6}$	Symptomatic infectious duration if not quarantined	1 + 4 = 7 days				^19^
$d+{d}_{4}$	Symptomatic infectious duration if quarantined	1 + 2 = 3 days				^19^
${d}_{5}$	Duration in quarantine before going to H, or ICU or D	5 days				
${v}_{1}$	Vaccine effectiveness against infection following two doses	5.9% AstraZeneca (AZ) 34.2% Pfizer following 6 months from vaccination and 88% for the newly vaccinated (just the 5-16)				^21^
${v}_{2}$	Vaccine effectiveness against infection following booster dose	75.5% Pfizer and 71.4%% AZ (60 + are considered to have AZ)				^21^
$N$	Total population	43,284,747				^28^
g	Asymptomatic	35%				^30^
H, hv1, hv2	Age specific hospitalization rates by vaccination status H = no vaccination hv1 = two doses hv2 = three doses	Age 0–4 5–9 10–14 15–19 20–29 30–49 50–59 60–69 70–74 75 +	H 0.69% 0.34% 0.45% 0.86% 1.37% 2.06% 3.25% 4.45% 7.54% 8.56%	Hv1 0.24% 0.12% 0.16% 0.31% 0.49% 0.73% 1.16% 1.59% 2.69% 3.06%	Hv2 0.17% 0.09% 0.11% 0.21% 0.34% 0.51% 0.81% 1.11% 1.88% 2.14%	^23^
icu, icuv1, icuv2	Age specific ICU rates by vaccination status icu = no vaccination icuv1 = two doses icuv2 = three doses	Age 0–4 5–9 10–14 15–19 20–29 30–49 50–59 60–69 70–74 75 +	icu 0.05% 0.05% 0.05% 0.11% 0.15% 0.15% 0.61% 1.06% 2.13% 1.37%	icuv1 0.01% 0.01% 0.01% 0.03% 0.04% 0.04% 0.15% 0.26% 0.51% 0.33%	icuv2 0.01% 0.01% 0.01% 0.02% 0.02% 0.02% 0.1% 0.17% 0.34% 0.22%	^23^
mu, muv1, muv2	Age specific deaths rates by vaccination status mu = no vaccination muv1 = two vaccination muv3 = three vaccination	Age 0–4 5–9 10–14 15–19 20–29 30–49 50–59 60–69 70–74 75 +	mu 0.02% 0.02% 0.02% 0.05% 0.12% 0.12% 0.12% 0.23% 0.94% 2.34%	muv1 0.01% 0.01% 0.01% 0.01% 0.03% 0.03% 0.03% 0.06% 0.25% 0.62%	muv2 0.01% 0.01% 0.01% 0.01% 0.03% 0.03% 0.03% 0.05% 0.22% 0.55%	^23^
dh	Duration in hospital	3 days for < 50 4.5 for 50-75 5 for 75 +				^25^
dicu	Duration in ICU	4 days				
H maximum capacity	Hospital beds in Ukraine	293,446 beds				^26^
m	Masks effectiveness in infection reduction	60% (40% tested for poor quality masks)				^27^
mr	Movement restriction	Varied				^17,18^

The median length of hospital stay was assumed to be 3, 4.5, and 5 days respectively for people < 50, 50–79, and 80 + years old^25^, and ICU stay is assumed to be 4 days. The total number of hospital beds in Ukraine came from a 2018 estimation of 293,446 beds^26^. This is a maximal estimate that does not account for the loss of hospital infrastructure and staffing during the war.

The implementation of mandatory mask use has been estimated to be 60% effective in reducing transmission if a combination of cloth and surgical masks is used^27^. If mask coverage is 50% and mask effectiveness is 60%, then using masks in Ukraine is estimated to be 30% effective^27^. We estimate that there may be 50% of people wearing masks at any one time as a base case scenario, based on 60% effectiveness, when fitting the model output to data. Mask use is implemented in the model with a reduction in the force of infection by the combination of the proportion of the population wearing it and mask effectiveness. We assume masks, if supplied, can be distributed to the population, but there is no data on the supply chain and logistics for medical items in Ukraine. We used the total population and age distribution from 2022 from Ukraine^28^ and an age-specific contact matrix from Ukraine^29^, where we used contact rates estimated for “all locations”. Then we used case and death notification data from the 6th of January 2022 to the 25th of February when Ukraine stopped reporting them^1^. We chose to start from the 6 of January as it is the start of the fourth wave attributed to the Omicron variant. As of this date, there were 4711 new cases notified, and as testing was only being done for symptomatic people, we considered this to represent 65% of the cases on that day, based on an estimated 35% of cases being asymptomatic^30^. Therefore, the total number of new infections on that day is estimated to be 7247 and we assumed the non-notified (2536) cases were infectious. The case notification data were fitted with the modelled case incidence using the estimation of restrictions from the Blavatnik School of Government restrictions tracker^17,18^. The nine metrics used to calculate the Stringency Index are school closures; workplace closures; cancellation of public events; restrictions on public gatherings; closures of public transport; stay-at-home requirements; public information campaigns; restrictions on internal movements; and international travel controls. The index on any given day is calculated as the mean score of the nine metrics. Movement restrictions are reflected in the model as a reduction of the contact rate. The model assumes that contact tracing, testing, and isolation of symptomatic people are not occurring or occurring at low levels. The restriction index for Ukraine was estimated to be 63% between the 6th and 17th of January (first pre-war period), then from the 18th of January to 25th of February it increased to 75% (second pre-war period), with the addition of the use of face masks in any public space and the closure of public transport. Those percentages were used in the model as the proportion of contacts reduced in those periods. The change from 63% to 75% restriction was gradually implemented in the model from the 18th of January to the first of February, to allow some adjustment time in the population to the new restrictions. From the 18th of January, the Ukraine policy changed from testing anyone to testing only symptomatic people^17,18^. We implemented this policy change in the model reducing the proportion of contact tracing from 60% to 20%, and then further reduced it to zero after the 26th of February when the war began. The vaccination rates as of 25 February, when last reported^1^ were 34.5% of the total population with 2 doses and only 1.7% with 3 doses. We distributed vaccine coverage with two doses to the 15 + age group (39.3% vaccinated) while three doses coverage is distributed only in the 60 + age group (6.3% vaccinated), following the policy for vaccination distribution. All the parameters used in the model are listed in Table 1.

### Model outputs

Firstly, we tested the model fit to notification data of cases, using the estimated restrictions from the 6^th^ of January to the 25^th^ of February. The model used overall death rates reported from European and US studies, of 0.3%, 0.08%, and 0.07% in unvaccinated, vaccinated with two and three doses, respectively. In this period, we assumed 50% of mask use and 60% to 20% of contact tracing (as detailed in Table 1) while estimating the reduction of contacts (movement restrictions). Then we forecast the epidemic curve. After the 25th of February, we assumed a small reduction (of 20%) in movements due to the unstable situation and people sheltering in place. This is because the number of contacts between different people would drastically be reduced, however, there is no data available on the impact of war on contact patterns. The model accounted for uncertainty with sensitivity analyses on mask use coverage and vaccination coverage. The base case scenario uses the last notified vaccination coverage and assumes 50% of people wearing masks. Results are shown with varying assumptions about mask use (0%, 50%, 80%) and varying vaccination rates from 39.3% (2 doses) and 6.3% (3 doses) as the base case, to 60% and 9.6%, and 80% and 12.8% respectively vaccinated with two and three doses in 15 + and 60 + age group. We included the scenario of 0% mask use to then estimate the averted cases when masks are used and show results with no mask use. We also tested decreasing mask effectiveness from 60% to 40% in the case of only poor-quality masks being available.

## Results

Figure 1 shows the modelled epidemic around the time of the start of the war in early 2022 fitted to observed data to estimate the reduction in population contacts. In the base case scenario where masks are 60% effective, the best fit to data shows a reduction in contacts of 33% in the first period and 47% to 70% in the second period (Fig. 1). Otherwise, in the case of masks being effective at 40%, the best fit resulted in a reduction in contacts of 40% in the first period and 53% to 73% in the second period.

**Figure 2:**
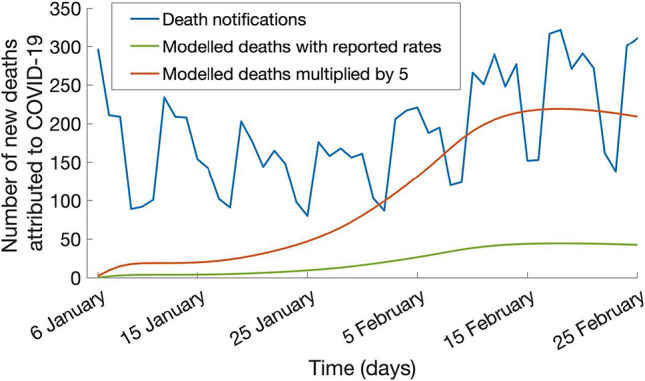
Modelled deaths (green line), multiplied by 5 (red line), and death notification data (blue line) between 6 January 2022 and 25 February 2022.

The modelled incidence of deaths in the same period, using reported rates from European and US studies, of 0.3%, 0.08%, and 0.07% in unvaccinated, vaccinated with two and three doses respectively (Fig. 2 green line), is much lower than rates reported in Ukraine (Fig. 2 blue line). Multiplying those numbers by 5 times, produces a much better fit (Fig. 2 red line), suggesting an under report of case numbers or higher death rates for Ukraine compared to the ones estimated in EU and the US used.

**Figure 3:**
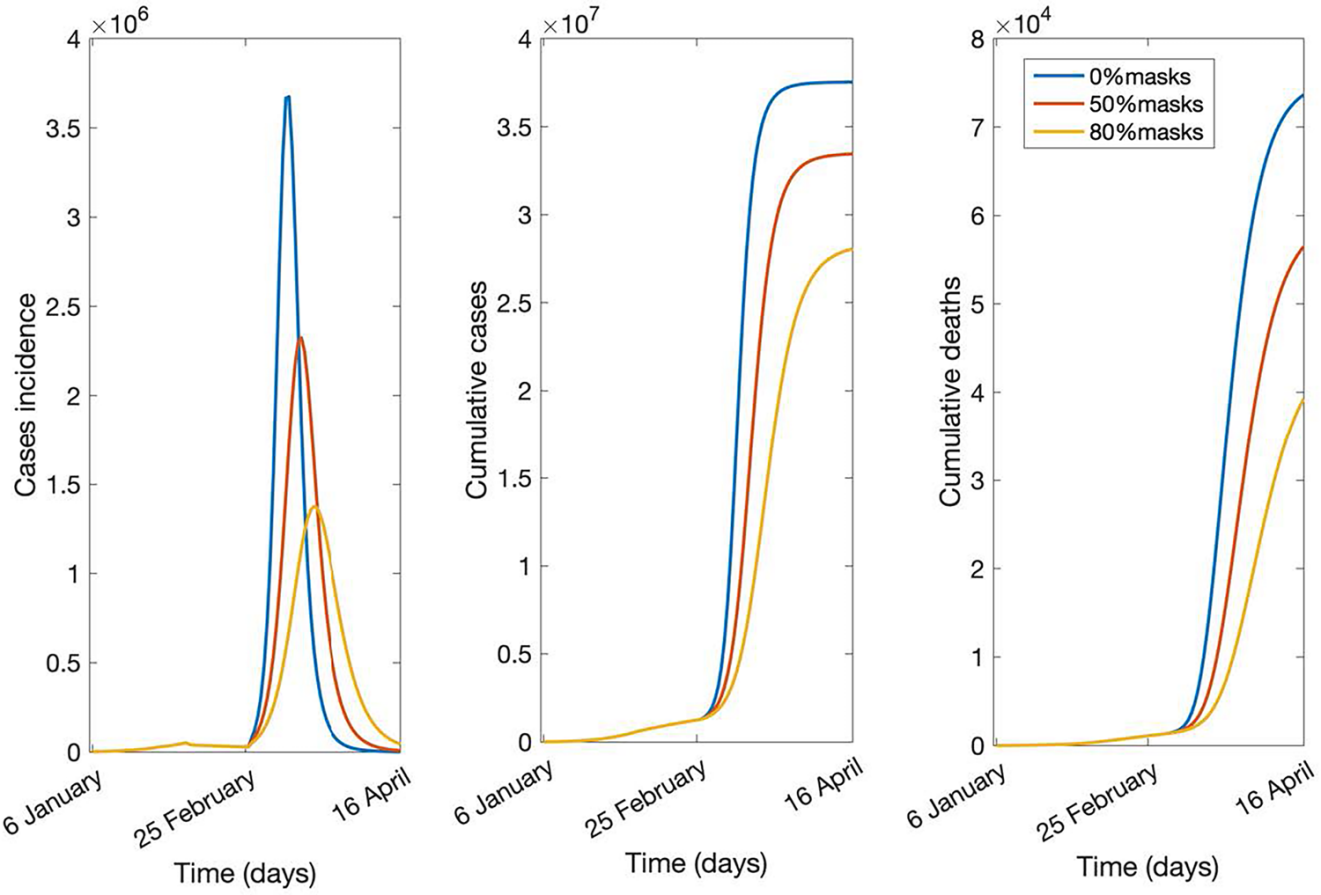
Number of daily new cases (incidence), cumulative cases, and cumulative deaths in the scenario with 39.3% of the 15 + age group with two doses and 6.3% of the 60 + with 3 doses, varying mask use coverage (0%, 50%, 80%) with 60% effectiveness, from 6 January to 14 April 2022.

**Figure 4:**
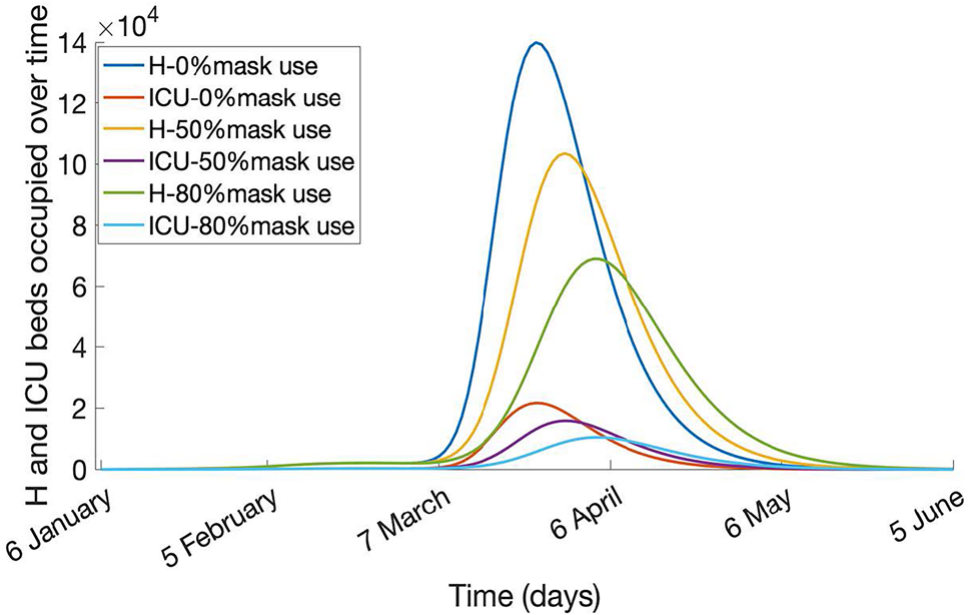
Hospitalization (H) and ICU beds used over time, keeping the last vaccination coverage notified (39.3% of the 15 + age group with two doses and 6.3% of the 60 + with 3 doses) and varying mask use coverage (0%, 50%, 80%), from 6 January to 5 June 2022.

The results of sensitivity analysis on mask use, based on the last reported vaccination rates (39.3% of the 15 + age group with two doses and 6.3% of the 60 + with 3 doses), are shown in Figs. 3 and 4. The epidemic forecast is shown in Fig. 3, with hospitalization and ICU daily bed requirements in Fig. 4. In each scenario, the epidemic peak was expected to be at the start of April 2022.

The outbreak peaks at about 3.7, 2.3, and 1.4 million cases, with a total of almost 90%, 80%, and 70% of the population being infected at the end of the outbreak, with 0%, 50%, and 80% of the population using masks, respectively (Fig. 3). Figure 4 shows that the maximum number of daily hospital beds required at the peak is estimated to be about 140, 103, and 69 thousand in scenarios of varying mask use, with a total of almost 300,000 beds available in Ukraine before the war started. The number of daily ICU beds required at the peak is estimated to be about 21, 15, and 10 thousand with 0%, 50%, and 80% mask use.

**Figure 5:**
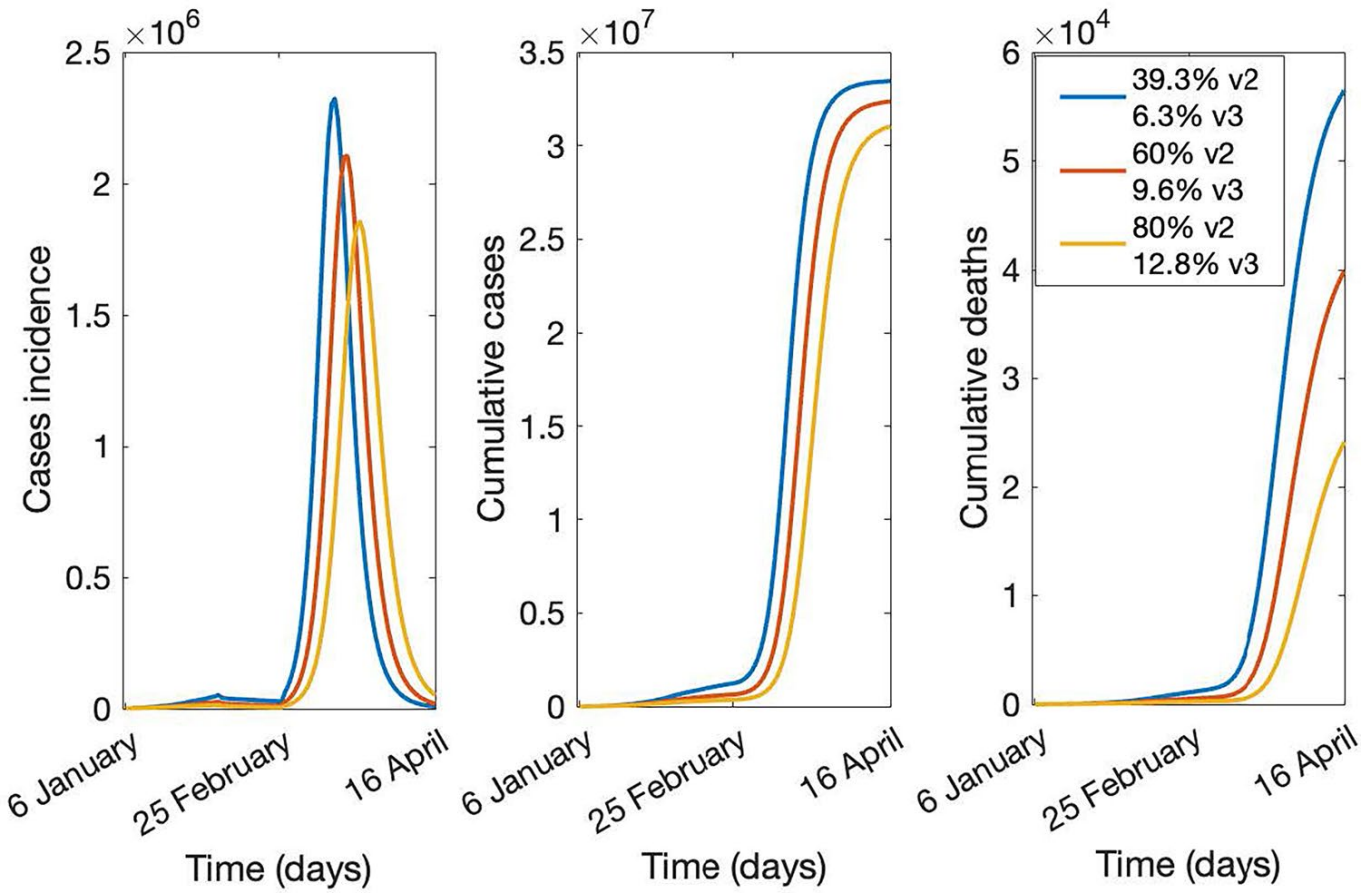
Case incidence, cumulative cases, and deaths in the scenario with 50% of the population using masks and varying the vaccination coverage, for 2 doses (v2) and three doses (v3), from 6 January to 16 April 2022.

**Figure 6:**
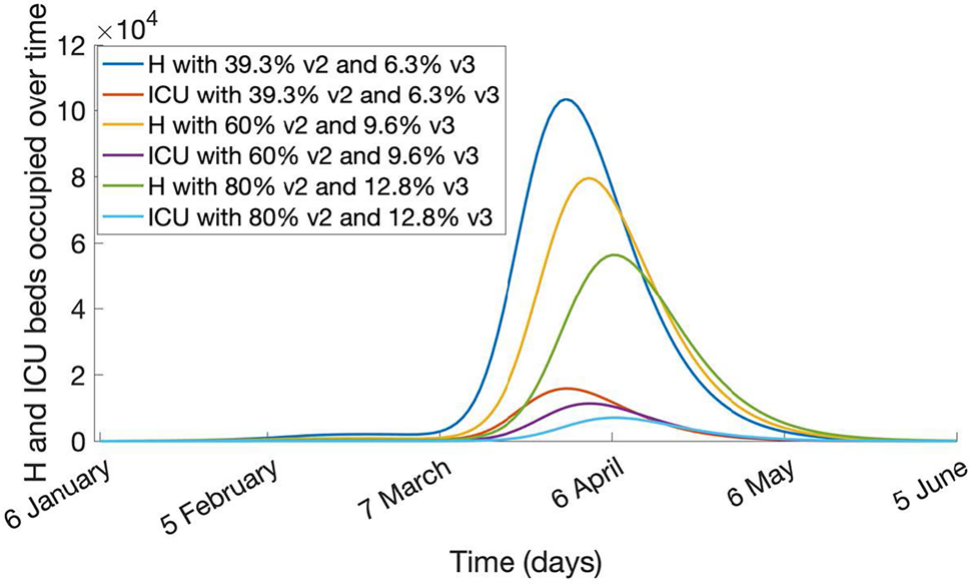
Hospitalization (H) and ICU beds used over time, with 50% of the population using masks and varying the vaccination coverage for two (v2) and three doses (v3), from 6 January to 5 June 2022.

The results of the sensitivity analysis on vaccination coverage are shown in Figs. 5 and 6. Figure 5 shows the epidemic forecast and Fig. 6, the hospitalization and ICU daily bed requirement with mask use at 50% and vaccination rates increased from 39.3% to 60% and 80%.

The outbreak peaks at about 2.3, 2.1, and 1.8 million cases, with a total of about 80%, 76%, and 74% of the population being infected at the end of the outbreak, at the three different vaccination scenarios (Fig. 5). The maximum number of hospital beds required at the peak is estimated to be about 103, 80, and 56 thousand in each scenario, while requirements for ICU beds have been estimated to be about 15, 11, and 7 thousand (Fig. 6).

From the base case scenario, the model shows increasing mask-wearing from 50% (base-case) to 80% could result in a 17% reduction in cases (from a total of 33,432,800 at 50% to 28,006,300 at 80%) and a 30% reduction in deaths (from 56,028 to 39,241). If vaccine coverage is increased from 39.3% and 6.3% with two and three doses respectively (base-case) to 60% of people aged 15 + with two doses and 9.6% of people aged 60 + with three doses, the reduction in cases and deaths could have been 3% (from 33,432,800 to 32,365,700) and 28% (from 56,028 to 39,867) respectively. However, when comparing the results of increasing mask use at 80% with the scenario where 80% and 12.8% of the population are vaccinated with two and three doses respectively, we found that high mask use results in a lower cumulative total number of cases (about 28 million) compared with high two doses vaccination coverage (about 31 million), but a higher number of total death (about 38,000 against 23,000). When testing if results were consistent in the case of only poor-quality masks (40% instead of 60% effectiveness) being available, we found that increasing mask use from 50% to 80% could have reduced cases and deaths by 6.1% (from 35,246,400 at 50% to 33,070,800 at 80% mask use) and 11.3% respectively (from 65,758 to 58,335). If vaccine coverage was increased to 60% with two doses and 9.6% of people aged 60 + with three doses, the reduction in cases and deaths could have been respectively 2.3% (from 35,246,400 to 34,443,600) and 25.6% (from 65,758 to 48,781).

## Discussion

### Results in context

This is the first study to quantify the effect of war on infectious disease transmission, and can inform public health response and COVID-19 outbreak control programs in Ukraine and neighboring countries accepting Ukrainian refugees.

With low vaccination rates and poor prospects of resuming vaccination campaigns during the war, the COVID-19 epidemic curve in Ukraine was expected to peak in April 2022 based on our estimates, with almost half of all available hospital beds required on the day of the peak. The availability of beds may be much less than the pre-war estimates due to the destruction of hospitals and the lack of healthcare staff who have either fled, are sheltering in place, or have suffered injury or illness. The case fatality rate from COVID-19 will rise if people who require hospital care, even the simplest of care such as oxygen therapy, are unable to receive it. In fact, our results suggest that even pre-war, Ukraine was seeing a higher than expected death rate from COVID-19, and this may reflect low health system capacity. Studies from Europe showed that when hospitals are overwhelmed and ICU occupancy is high, case fatality rates rise^31^.

We showed that increasing vaccination rates to 60% is less effective than increasing mask use to 80% in reducing transmissions, largely due to the low efficacy of two doses against transmission. However, if vaccination coverage is increased to 80% or mask effectiveness is reduced because of low-quality masks being available, increasing vaccination coverage is more effective in reducing the number of deaths.

### Study limitations

There are several limitations in this study. One is the reliability of data. We showed a good fit of our model to the case notifications data, but the deaths in Ukraine are much higher than estimated in the rest of Europe^22^ and the US^23^ for the Omicron variant. The modelled death rates were lower than the observed data, and a good fit is seen only when multiplying those rates by 5 times, suggesting a high proportion of unnotified COVID-19 cases, or higher death rates due to a combination of low vaccination rates and lack of hospital care for the critically ill in Ukraine compared to the rates used estimated for EU and the US.

We assumed the mask effectiveness to be 60%^27^, however, the effectiveness varies depending on the type of masks used, therefore we show results for poor-quality masks. One of the strengths of this study is the use of age-specific parameters and contacts. However, another limitation, highlighted already from a previous similar modelling study of COVID-19 in Ukraine using the same contacts matrix^32^, is that we did not account for variation between different Ukrainian regions in terms of their population structure, local transportation modes, patterns of work and other factors which affect disease spread in those regions^32^. Furthermore, there is no data on the impact of war on contact patterns, hospitalization, and death rates. The hospitalization and death rates previously estimated for the Delta variant in Ukraine^32^ are much higher than the ones used here, adapted for the Omicron variant. However, health system disruption, which could result in a large increase in death rates, is not considered in this study and this could show an underestimation of the disease burden. The relative difference between Omicron and Alpha or D614G in an unvaccinated population is less clear. Studies suggest Delta is twice as severe as Alpha, so Omicron may be similar to Alpha in the unvaccinated^33^. Furthermore, we assumed vaccine effectiveness against Omicron infection to be the one estimated for Astra Zeneca and Pfizer. However, 30% to 40% of the Ukraine population received a different vaccine for the first two doses which may had lower effectiveness. The impact of vaccination, therefore, may be less than the model suggests. Following three doses, we used a vaccine effectiveness of over 70%, however, it has been shown that after two months from vaccination, this effectiveness may wanes to 47%, therefore we could have overestimated the benefit of having increased vaccine coverage on the case incidence^7^. Vaccine effectiveness against hospitalization and ICU admission is uncertain, as some studies show consistent effectiveness in time^7^ and others show rapid waning^6^. We did not consider reinfection, as the model only runs for 150 days, and data at that time on re-infection was limited. Many other, more immune-evasive variants of Omicron have emerged in the two years since the war began, which may further reduce the impact of vaccination.

### Comparison with a country not affected by war with a good working health system

Starting from a low baseline of 34.5% and 1.7% of the total population with two and three doses of coverage of COVID-19 vaccination, Ukraine was highly vulnerable to COVID-19 at the time of the Russian invasion of the country. We estimated that in the first half of 2022, in the best-case scenario with 80% of the population wearing masks, about 69% of the Ukrainian population (27.5 million) could have been infected with COVID-19 with almost 70,000 beds needed per day at the peak, and a total of about 28,000 cumulative deaths. Contrasting the situation in Ukraine with a country where there is peace and high vaccination coverage is informative. Australia is such a country, which rapidly achieved over 95% two-dose coverage and was using masks and other mitigations such as antivirals early 2022. In Australia it is estimated that 42% of the population (10.9 million) have been infected^34^ with less than 9000 deaths registered in 2022^35,36^, with hospital bed requirements of 1300 per day at the peak in January, and not exceeding 700 per day after January 2022^37^. The population of Ukraine is just under double that of Australia. The estimated death rate for 2022 in Australia was about 0.08%, while in Ukraine, with lower vaccination rates, is estimated to be 0.16%. Hospitalization beds required at the peak in Ukraine were orders of magnitude higher, with 0.24% of the population being hospitalized at the peak compared with 0.005% in Australia.

## Conclusions and recommendations

In the context of conflict, COVID-19 could result in a much higher number of deaths, due to poor public health infrastructure, disruption of health systems, and interruption to vaccination programs. Reduced access to hospital or ICU beds, likely resulted in higher case fatality^38–41^. Our study shows that masks can reduce the impact of COVID-19 in Ukraine. If the cold chain cannot be maintained, health systems are unable to function and vaccines cannot be distributed, masks may be a more feasible and effective way to mitigate the epidemic during war. Indeed, masks can be distributed without functioning health infrastructure.

Vaccination campaigns and the provision of high-quality masks, together with the provision of health clinics and workforce, should be part of any humanitarian response to the situation in Ukraine^38–41^. Furthermore, since the start of the war on 24 February 2022, several millions of people have fled the country, and vaccination of refugees should be a high priority and may be more feasible than delivering vaccine programs in-country^42^.

### Supplementary Information

Supplementary Information.

## Data Availability

The datasets generated and/or analysed during the current study are available online, [Coronavirus cases Ukraine—COVID Live [Internet], [cited 2022 Mar 18]. Available from: https://covidlive.com.au/country/ukraine].
