# Supplementary material for: Impact of vaccine coverage and disruption to health services on COVID-19 in Ukraine
Source: Sci Rep. 2024 Jun 26;14:14729. doi: 10.1038/s41598-024-57447-7 (PMC11208616; doi:10.1038/s41598-024-57447-7)
Supplement: Supplementary file 1 — Supplementary Information. [file 41598_2024_57447_MOESM1_ESM.docx]

**Appendix**

**Section 1. Model description**

The differential equations move the population through disease epidemiological stages and response stages (Figure 1S). The epidemiological stages that people move through are: being susceptible, not vaccinated (S), vaccinated with two doses (V1) or three doses (V2), latent not infectious yet (E and Ev1 and Ev2), latent infectious undiagnosed (E^u^, Ev1^u^, Ev2^u^) and diagnosed (E^t^, Ev1^t^, Ev2^t^), first symptomatic infectious day stages for undiagnosed (I1^u^, I1v1^u^, and I1v2^u^) and diagnosed (I1^t^, I1v1^t^, and I1v2^t^), following symptomatic infectious days for undiagnosed (I2^u^, I2v1^u^, and I2v2^u^) and diagnosed (I2^t^, I2v1^t^, and I2v2^t^), recovered (R) or death (D). The public health response stages are being isolated (Q, Qv1, and Qv2), cases hospitalized (H), and requiring intensive care unit (ICU). The model has two compartments for asymptomatic people who never develop symptoms, for the first day and following 6 days of symptomatic infectious respectively, A1 and A2. Each of those compartments is age-specific (i) for 16 age groups, 5 years wide 0-74 years old, and a 75+ last age group.


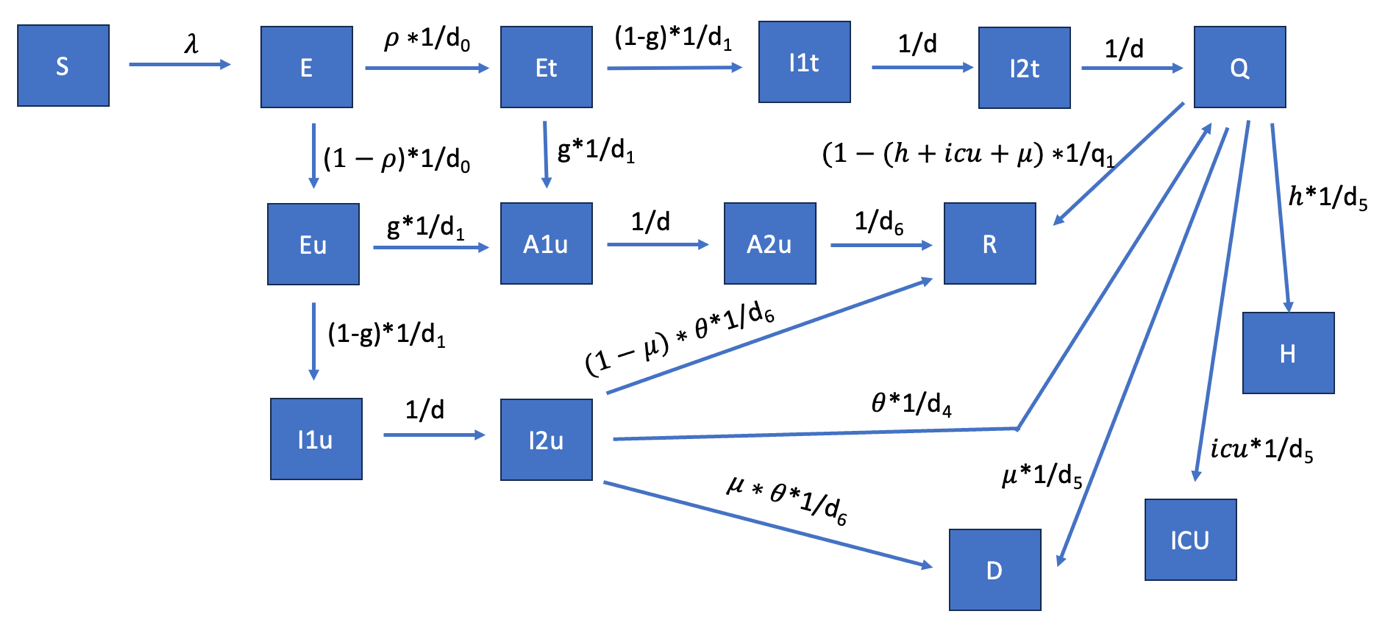


Figure 1S: Model diagram, each of those compartments is age-specific (i), and every compartment apart from the final ones (R, H, ICU, D) is divided by vaccination status.

**Section 2. Differential equations**

$${dS}_{i}/dt=-\lambda*S_{i}$$

$$\frac{{dV1}_{i}}{dt}= - (1-v1)*\lambda*{V1}_{i}$$

$${dV2}_{i}/dt=-(1-v2)*\lambda*{V2}_{i}$$

$$dE_{i}/dt=\lambda*S_{i}-E_{i}/d_{0}$$

$$d{Ev1}_{i}/dt=(1-v1)*\lambda*{V1}_{i}-{Ev1}_{i}/d_{0}$$

$$d{Ev2}_{i}/dt=(1-v2)*\lambda*{V2}_{i}-{Ev2}_{i}/d_{0}$$

$${dE}_{i}^{u}/dt=(1-\rho)*E_{i}/d_{0}-E_{i}^{u}/d_{1}$$

$${dEv1}_{i}^{u}/dt=(1-\rho)*{Ev1}_{i}/d_{0}-{Ev1}_{i}^{u}/d_{1}$$

$${dEv2}_{i}^{u}/dt=(1-\rho)*{Ev2}_{i}/d_{0}-{Ev2}_{i}^{u}/d_{1}$$

$${dE}_{i}^{t}/dt=\rho*E_{i}/d_{0}-E_{i}^{t}/d_{1}$$

$${dEv1}_{i}^{t}/dt=\rho*{Ev1}_{i}/d_{0}-{Ev1}_{i}^{t}/d_{1}$$

$${dEv2}_{i}^{t}/dt=\rho*{Ev2}_{i}/d_{0}-{Ev2}_{i}^{t}/d_{1}$$

$${dI1}_{i}^{u}/dt={(1-g)*E}_{i}^{u}/d_{1}-{I1}_{i}^{u}/d$$

$${dI1v1}_{i}^{u}/dt={(1-g)*Ev1}_{i}^{u}/d_{1}-{I1v1}_{i}^{u}/d$$

$${dI1v2}_{i}^{u}/dt={(1-g)*Ev2}_{i}^{u}/d_{1}-{I1v2}_{i}^{u}/d$$

$${dI1}_{i}^{t}/dt={(1-g)*E}_{i}^{t}/d_{1}-{I1}_{i}^{t}/d$$

$${dI1v1}_{i}^{t}/dt={(1-g)*Ev1}_{i}^{t}/d_{1}-{I1v1}_{i}^{t}/d$$

$${dI1v2}_{i}^{t}/dt={(1-g)*Ev2}_{i}^{t}/d_{1}-{I1v2}_{i}^{t}/d$$

$${dA1}_{i}^{u}/dt=g*(\frac{E_{i}^{u}}{d_{1}}+\frac{E_{i}^{t}}{d_{1}}+\frac{{Ev1}_{i}^{u}}{d_{1}}+\frac{{Ev1}_{i}^{t}}{d_{1}}+\frac{{Ev2}_{i}^{u}}{d_{1}}+\frac{{Ev2}_{i}^{t}}{d_{1}}) -{A1}_{i}^{u}/d$$

$${dI2}_{i}^{u}/dt={I1}_{i}^{u}/d-\theta*{I2}_{i}^{u}/d_{4}-(1-\theta) *{I2}_{i}^{u}/d_{6}$$

$${dI2v1}_{i}^{u}/dt={I1v1}_{i}^{u}/d-\theta*{I2v1}_{i}^{u}/d_{4}-(1-\theta) *{I2v1}_{i}^{u}/d_{6}$$

$${dI2v2}_{i}^{u}/dt={I1v2}_{i}^{u}/d-\theta*{I2v2}_{i}^{u}/d_{4}-(1-\theta) *{I2v2}_{i}^{u}/d_{6}$$

$${dI2}_{i}^{t}/dt={I1}_{i}^{t}/d-{I2}_{i}^{t}/d$$

$${dI2v1}_{i}^{t}/dt={I1v1}_{i}^{t}/d-{I2v1}_{i}^{t}/d$$

$${dI2v2}_{i}^{t}/dt={I1v2}_{i}^{t}/d-{I2v2}_{i}^{t}/d$$

$${dA2}_{i}^{u}/dt={A1}_{i}^{u}/d- {A2}_{i}^{u}/d_{6}$$

$${dQ}_{i}/dt=\frac{{I2}_{i}^{t}}{d}+\theta*\frac{{I2}_{i}^{u}}{d_{4}} -(1-(h+icu+\mu) )*Q_{i}/q_{1} -(h+icu+\mu) *Q_{i}/d_{5}$$

$${dQv1}_{i}/dt=\frac{{I2v1}_{i}^{t}}{d}+\theta*\frac{{I2v1}_{i}^{u}}{d_{4}} -(1-(hv1+icuv1+\mu v1) )*{Qv1}_{i}/q_{1} -(hv1+icuv1+\mu v1) *{Qv1}_{i}/d_{5}$$

$${dQv2}_{i}/dt=\frac{{I2v2}_{i}^{t}}{d}+\theta*\frac{{I2v2}_{i}^{u}}{d_{4}} -(1-(hv2+icuv2+\mu v2) )*{Qv2}_{i}/q_{1} -(hv2+icuv2+\mu v2) *{Qv2}_{i}/d_{5}$$

$${dH}_{i}/dt=h *Q_{i}/d_{5}+hv1 *{Qv1}_{i}/d_{5}+hv2 *{Qv2}_{i}/d_{5}-H_{i}/dh$$

$${dICU}_{i}/dt= icu*Q_{i}/d_{5}+{icuv1*\frac{{Qv1}_{i}}{d_{5}}+icuv2*\frac{{Qv2}_{i}}{d_{5}}-ICU}_{i}/dicu$$

$${dR}_{i}/dt=\left( 1-\mu_{i} \right)*\left( 1-\theta\right)*{I2}_{i}^{u}/d_{6}+{\left( 1-{\mu v1}_{i} \right)*\left( 1-\theta\right)*{I2v1}_{i}^{u}/d_{6}+\left( 1-{\mu v2}_{i} \right)*\left( 1-\theta\right)*{I2v2}_{i}^{u}/d_{6}+A2}_{i}^{u}/d_{6}+\left( 1-\left( h+icu+\mu\right) \right)*\frac{Q_{i}}{q_{1}}+\left( 1-\left( hv1+icuv1+\mu v1 \right) \right)*\frac{{Qv1}_{i}}{q_{1}}+ (1-(hv2+icuv2+\mu v2) )*{Qv2}_{i}/q_{1}+H_{i}/dh +{ICU}_{i}/dicu$$

$${dD}_{i}/dt=\mu_{i}*\left( 1-\theta\right)*{I2}_{i}^{u}/d_{6}+{\mu v1}_{i}*\left( 1-\theta\right)*{I2v1}_{i}^{u}/d_{6}+{\mu v2}_{i}*\left( 1-\theta\right)*{I2v2}_{i}^{u}/d_{6}+\mu_{i}*Q_{i}/d_{5}+{\mu v1}_{i}*{Qv1}_{i}/d_{5}+{\mu v2}_{i}*{Qv2}_{i}/d_{5}$$

The force of infection is described as

$$\lambda_{i}=\sum_{j=1}^{18} \frac{\beta_{1}*c_{i,j}*{(E}_{j}^{u}+{Ev1}_{j}^{u}+{Ev2}_{j}^{u})}{N}+\sum_{j=1}^{18} \frac{\beta_{2}*c_{i,j}*{(E}_{j}^{t}+{Ev1}_{j}^{t}+{Ev2}_{j}^{t})}{N}+\sum_{j=1}^{18} \frac{\beta_{3}*c_{i,j}*{(I1}_{j}^{u}+{I1v1}_{j}^{u}+{I1v2}_{j}^{u}+{A1}_{j}^{u})}{N}+\sum_{j=1}^{18} \frac{\beta_{4}*c_{i,j}*{(I1}_{j}^{t}+{I1v1}_{j}^{t}+{I1v2}_{j}^{t})}{N}$$

$$+\sum_{j=1}^{18} \frac{\beta_{5}*c_{i,j}*{(I2}_{j}^{u}+{I2v1}_{j}^{u}+{I2v2}_{j}^{u}+{A2}_{j}^{u})}{N}+\sum_{j=1}^{18} \frac{\beta_{6}*c_{i,j}*{(I2}_{j}^{t}+{I2v1}_{j}^{t}+{I2v2}_{j}^{t})}{N}$$

Where $\beta_{i}$ is the probability of transmission per contact, for pre-symptomatic untraced and traced, first day of symptoms untraced and traced and following 6 days after symptoms onset for untraced and traced respectively. The infectious period is divided in 2 pre-symptomatic days and 7 symptomatic days, where each stage of traced infectious has 50% reduction in transmissions.$c_{i,j}$ is the age-specific contact matrix estimated for Ukraine, and *N* is the total population. Then we added the reduction in transmission by mask use (multiplied $\lambda_{i}$ by $1-m_{i}$) where $m_{i}$ is a combination of proportion of the population wearing it and mask effectiveness to reduce the force of infection. Movement restrictions are reflected in the model as a reduction of the contact rate.

All the parameters used are in Table 1

**Section 3. Table 1: Model parameters**

| **Symbol** | **Definition** | **Value** | **Source** |
| --- | --- | --- | --- |
| ***R0*** | Basic reproductive number | 6 | (1–3) |
| $\boldsymbol{\theta}$ | Percentage of symptomatic people isolated | 90% | (4) |
| **ρ** | Contacts traced for each case | 60% (6-17 January)  45% (18 January-5 February)  20% (6-25 February)  0% following the 25 February | (5,6) |
| $\boldsymbol{q}_{\boldsymbol{1}}$ | Duration of quarantine for contacts traced isolation for symptomatic | 7 |  |
| $\boldsymbol{d}_{\boldsymbol{0}}$ | Latent duration not infectious | 1 day |  |
| ***d*** | Duration in Infectious period before being traced | 1 day |  |
| $\boldsymbol{d}_{\boldsymbol{1}}$ | Pre symptomatic infectious duration | 2 days | (4) |
| $\boldsymbol{d+}\boldsymbol{d}_{\boldsymbol{6}}$ | Symptomatic infectious duration if not quarantined | 1+6=7 days | (4) |
| $\boldsymbol{d+}\boldsymbol{d}_{\boldsymbol{4}}$ | Symptomatic infectious duration if quarantined | 1+2=3 days | (4) |
| $\boldsymbol{d}_{\boldsymbol{5}}$ | Duration in quarantine before going to H, or ICU or D | 5 days |  |
| $\boldsymbol{v}_{\boldsymbol{1}}$ | Vaccine effectiveness against infection following two doses | 5.9% AstraZeneca (AZ)  34.2% Pfizer following 6 months from vaccination and 88% for the newly vaccinated (just the 5-16) | (7) |
| $\boldsymbol{v}_{\boldsymbol{2}}$ | Vaccine effectiveness against infection following booster dose | 75.5% Pfizer and 71.4%% AZ  (60+ are considered to have AZ) | (7) |
| $\boldsymbol{N}$ | Total population | 43,284,747 | (8) |
| **g** | Asymptomatic | 35% | (9) |
| **H, hv1, hv2** | Age specific hospitalization rates by vaccination status  H=no vaccination  hv1=two doses  hv2=three doses | \| Age  0-4  5-9  10-14  15-19  20-29  30-49  50-59  60-69  70-74  75+ \| H  0.69%  0.34%  0.45%  0.86%  1.37%  2.06%  3.25%  4.45%  7.54%  8.56% \| Hv1  0.24%  0.12%  0.16%  0.31%  0.49%  0.73%  1.16%  1.59%  2.69%  3.06% \| Hv2  0.17%  0.09%  0.11%  0.21%  0.34%  0.51%  0.81%  1.11%  1.88%  2.14% \| \| --- \| --- \| --- \| --- \| | (10) |
| **icu, icuv1, icuv2** | Age specific ICU rates by vaccination status  icu=no vaccination  icuv1=two doses  icuv2=three doses | \| Age  0-4  5-9  10-14  15-19  20-29  30-49  50-59  60-69  70-74  75+ \| icu  0.05%  0.05%  0.05%  0.11%  0.15%  0.15%  0.61%  1.06%  2.13%  1.37% \| icuv1  0.01%  0.01%  0.01%  0.03%  0.04%  0.04%  0.15%  0.26%  0.51%  0.33% \| icuv2  0.01%  0.01%  0.01%  0.02%  0.02%  0.02%  0.1%  0.17%  0.34%  0.22% \| \| --- \| --- \| --- \| --- \| | (10) |
| **mu, muv1, muv2** | Age specific deaths rates by vaccination status  mu=no vaccination  muv1=two vaccination  muv3=three vaccination | \| Age  0-4  5-9  10-14  15-19  20-29  30-49  50-59  60-69  70-74  75+ \| mu  0.02%  0.02%  0.02%  0.05%  0.12%  0.12%  0.12%  0.23%  0.94%  2.34% \| muv1  0.01%  0.01%  0.01%  0.01%  0.03%  0.03%  0.03%  0.06%  0.25%  0.62% \| muv2  0.01%  0.01%  0.01%  0.01%  0.03%  0.03%  0.03%  0.05%  0.22%  0.55% \| \| --- \| --- \| --- \| --- \| | (10) |
| **dh** | Duration in hospital | 3 days for <50  4.5 for 50-75  5 for 75+ | (11) |
| **dicu** | Duration in ICU | 4 days |  |
| **H maximum capacity** | Hospital beds in Ukraine | 293,446 beds | (12) |
| **m** | Masks effectiveness in infection reduction | 60% (40% tested for poor quality masks) | (13) |
| **mr** | Movement restriction | Varied | (5,6) |

**References**

1. Lusvarghi S, Pollett SD, Neerukonda SN, Wang W, Wang R, Vassell R, et al. SARS-CoV-2 BA.1 variant is neutralized by vaccine booster-elicited serum, but evades most convalescent serum and therapeutic antibodies. Sci Transl Med. 2022 Apr 5;

2. Regev-Yochay G, Gonen T, Gilboa M, Mandelboim M, Indenbaum V, Amit S, et al. Efficacy of a Fourth Dose of Covid-19 mRNA Vaccine against Omicron. New England Journal of Medicine. 2022 Apr 7;386(14):1377–80.

3. Tseng HF, Ackerson BK, Luo Y, Sy LS, Talarico CA, Tian Y, et al. Effectiveness of mRNA-1273 against SARS-CoV-2 Omicron and Delta variants. Nature Medicine 2022. 2022 Feb 21;1–9.

4. MacIntyre CR, Costantino V, Trent M. Modelling of COVID-19 vaccination strategies and herd immunity, in scenarios of limited and full vaccine supply in NSW, Australia. Vaccine. 2021 Apr 24;

5. Mathieu E, Ritchie H, Ortiz-Ospina E, Roser M, Hasell J, Appel C, et al. Coronavirus Pandemic (COVID-19). Our World in Data. 2020 Mar 5;5(7):947–53.

6. COVID-19 Government Response Tracker | Blavatnik School of Government [Internet]. [cited 2022 Mar 18]. Available from: https://www.bsg.ox.ac.uk/research/research-projects/covid-19-government-response-tracker

7. Andrews N, Stowe J, Kirsebom F, Toffa S, Rickeard T, Gallagher E, et al. Effectiveness of COVID-19 vaccines against the Omicron (B.1.1.529) variant of concern.

8. Ukraine Population 2022 (Demographics, Maps, Graphs) [Internet]. [cited 2022 Mar 18]. Available from: https://worldpopulationreview.com/countries/ukraine-population

9. Sah P, Fitzpatrick MC, Zimmer CF, Abdollahi E, Juden-Kelly L, Moghadas SM, et al. Asymptomatic SARS-CoV-2 infection: A systematic review and meta-analysis. Proc Natl Acad Sci U S A [Internet]. 2021 Aug 24 [cited 2022 May 16];118(34). Available from: https://doi.org/10.1073/pnas.2109229118

10. Danza P, Koo TH, Haddix M, Fisher R, Traub E, OYong K, et al. SARS-CoV-2 Infection and Hospitalization Among Adults Aged ≥18 Years, by Vaccination Status, Before and During SARS-CoV-2 B.1.1.529 (Omicron) Variant Predominance — Los Angeles County, California, November 7, 2021–January 8, 2022. MMWR Morb Mortal Wkly Rep. 2022;71(5):177–81.

11. Average hospital stay for Omicron patients to be a game-changer? Experts share data [Internet]. [cited 2022 Feb 15]. Available from: https://www.republicworld.com/technology-news/science/average-hospital-stay-for-omicron-patients-to-be-a-game-changer-experts-share-data-articleshow.html

12. Healthcare Resource Guide - Ukraine [Internet]. [cited 2024 Jan 12]. Available from: https://www.trade.gov/healthcare-resource-guide-ukraine

13. Andrejko KL, Pry JM, Myers JF, Fukui N, DeGuzman JL, Openshaw J, et al. Effectiveness of Face Mask or Respirator Use in Indoor Public Settings for Prevention of SARS-CoV-2 Infection — California, February–December 2021. MMWR Morb Mortal Wkly Rep. 2022 Feb 11;71(6):212–6.
